# Large Angle Optical Access in a Sub-Kelvin Cryostat

**DOI:** 10.1007/s10909-018-1940-1

**Published:** 2018-05-15

**Authors:** S. Hähnle, J. Bueno, R. Huiting, S. J. C. Yates, J. J. A. Baselmans

**Affiliations:** SRON Netherlands Institute for Space Research, 3584 CA Utrecht, Netherlands

**Keywords:** Cryostat, Lens-antenna, Kinetic inductance detectors, Cryogenic optics

## Abstract

The development of lens-antenna-coupled aluminum-based microwave kinetic inductance detectors (MKIDs) and on-chip spectrometers needs a dedicated cryogenic setup to measure the beam patterns of the lens-antenna system over a large angular throughput and broad frequency range. This requires a careful design since the MKID has to be cooled to temperatures below $300\,\hbox {mK}$ to operate effectively. We developed such a cryostat with a large opening angle $\theta = \pm \,37.8^\circ$ and an optical access with a low-pass edge at $950\,\hbox {GHz}$. The system is based upon a commercial pulse tube cooled 3 K system with a $^4\hbox {He}$–$^3\hbox {He}$ sorption cooler to allow base temperatures below $300\,\hbox {mK}$. A careful study of the spectral and geometric throughput was performed to minimize thermal loading on the cold stage, allowing a base temperature of $265\,\hbox {mK}$. Radio-transparent multi-layer-insulation was employed as a recent development in filter technology to efficiently block near-infrared radiation.

## Introduction

Microwave kinetic inductance detectors (MKIDs) [1] become an increasingly attractive option for large-scale imaging instruments [2]. An interesting alternative to lumped element MKIDs [3] are lens-antenna-coupled MKIDs [4], which couple radiation using a coherent beam formed by the lens-antenna system. The advantage is that they are sensitive to radiation over a limited angular throughput given by the lens-antenna design. Therefore, they reject large angle radiation, which allows the use of a higher temperature optics and Lyot stop. This results in cryogenically simple camera designs [5].

Imaging arrays of antenna-coupled MKIDs have fast beams to reduce the chip area for a given field of view: fast optics results in a spatially small airy pattern, smaller pixels and smaller arrays. The beam width of these antennas at the $-10$ dB taper is typically in the order of $\pm \,20^{\circ }$, which makes measurements of the antennas full beam pattern, including side lobes, challenging to implement. However, it is critical to measure the beam shape to fully understand the detector performance [6]. In this paper, we present a cryostat that is designed to enable large angular throughput beam pattern measurements for antenna-coupled MKIDs. It fulfills the following requirements:

- Sub-millimeter (sub-mm) wave access from the laboratory through a window with a large opening angle to the detector.
- No reimaging optics in order to measure the unperturbed beam pattern.
- Optical access to the detector over a spectral passband of up to 1 THZ.
- Fast cooldown speed of the cryostat and easy assembly process motivated by the fast turnaround speed (1–2 days) necessary for an efficient iterative antenna development.
- The cold stage temperature of less than 270 mK for operation of the detectors.

## System Overview

**Fig. 1:**
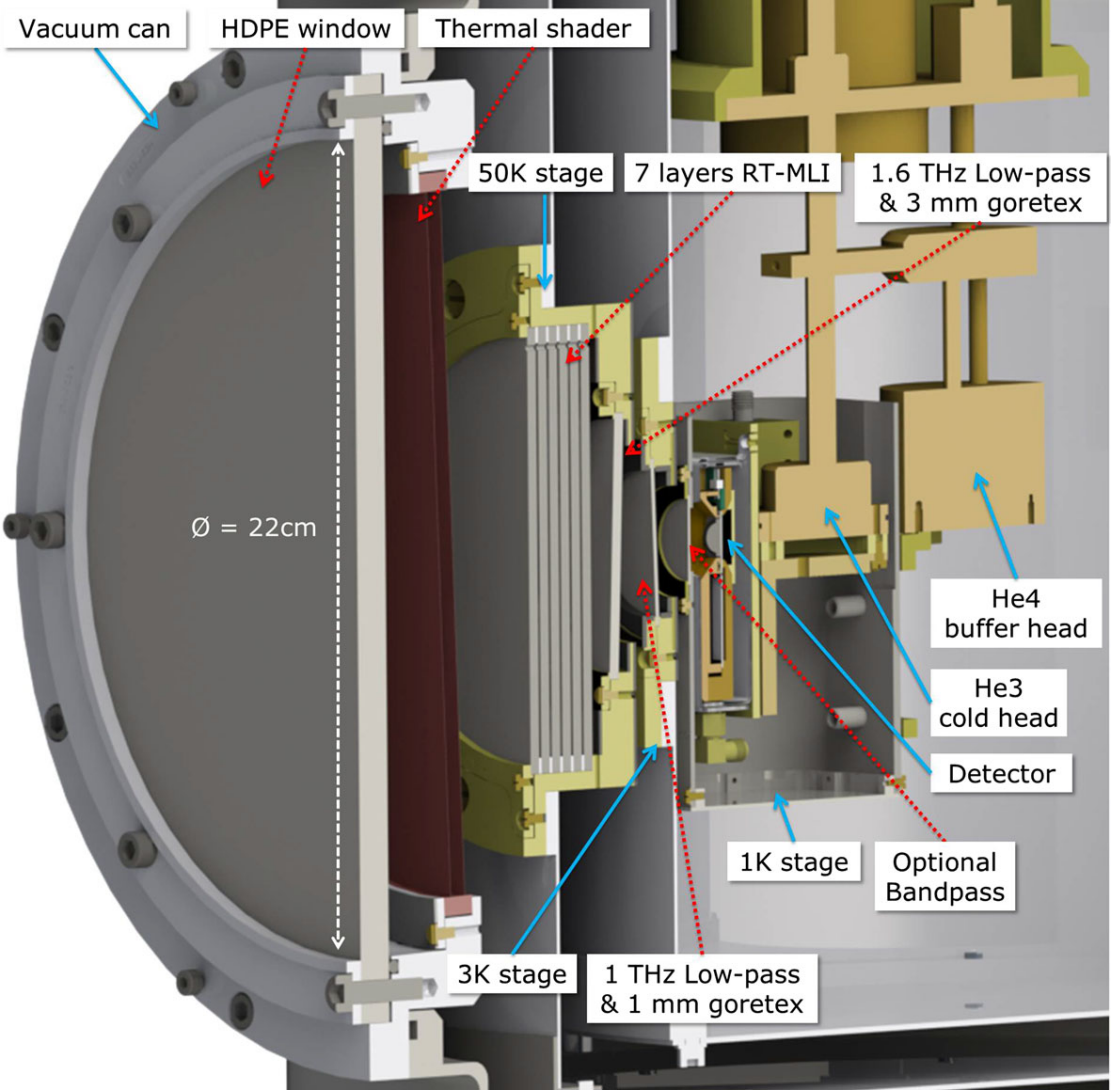
Cross section of the cryostat’s optical access, showing all windows including the filters. A 1-cm-thick polyethylene window in the vacuum can is air-tight but transparent in the sub-mm regime. In addition, a single thermal shader is mounted directly behind the vacuum window. The apertures in the 50 K and 3 K radiation shields are made of gold-plated copper and serve as mounting point for the filter stack: 7 layers of RT-MLI on the outer side of the 50 K window, as well as reflective metal mesh filters on both the 50 K and 3 K window. The latter are oriented at angles with respect to each other and the detector to avoid standing waves. Both the 1 K radiation shield and the detector assembly are located in tight proximity to the 3 K window, with the 1 K shield mounted on the $^4\hbox {He}$ buffer head and the detector assembly directly on the $^3\hbox {He}$ cold head. The 1 K shield is aluminum and serves as an optional mounting point for additional filter. A cryophy magnetic shield is integrated in the detector assembly (Color figure online)

A cross section of the optical access for the cryostat is shown in Fig. 1. The cryogenic system consists of standard commercially available components. A pulse tube cooled cryostat (BlueFors Cryogenics) provides 0.9 W cooling power at 4.2 K, where a sorption cooler (*CRC-7B-002*, Chase Research Cryogenics) is mounted that can reach temperatures down to 240 mK. The sorption cooler is a two-stage, single shot system consisting of a $^3\hbox {He}$ cold head as the mounting point for experiments and a $^4\hbox {He}$ buffer head.

The windows are in a cone-like configuration, providing a half opening angle of $\theta = 37.8^\circ$ to the center of a detector chip mounted on the cold stage of the sorption cooler. The design of the geometry of these apertures is critical to the cooler performance and will be discussed in detail in the next section. Access to the cold stage is possible by removing only the vacuum can and heat shields, which allows for fast and efficient operation of the system.

The detector assembly mounted on the cold stage can hold different chip dimensions although only an area of $(10 \times 10)\,\hbox {mm}^2$ is optically accessibly. Connection to room temperature electronics to read out the MKIDs is provided by means of a single pair of semirigid coax cables. From room temperature to the 3 K stage, 2.19-mm-diameter CuNi coax cables are used, while 0.86-mm-diameter and 15-mm-long NbTi cables (COAX CO., LTD.) provide a lossless connection from 3 K to the detector on the cold stage. The thermal load on the cold stage due to the coax cables is only $16\,\hbox {nW}$, which is negligible compared to radiative loading from the optical access. The readout signal is amplified at the 3 K stage using a commercial low-noise amplifier [7].

We design an infrared passband from 0 to 950 GHz by a combination of commercial metal mesh filters (QMC Instruments Ltd.), gore tex sheets and a RT-MLI infrared blocking assembly [8]. The combination of the optimized geometry and filter stack allows us to reach a loading on the cold stage of $6\, \upmu \hbox {W}$, while the total power entering the cryostat window is $17\,\hbox {W}$. The spectral filtering thus reduces the input power by 40 dB and the geometric baffling by another 30 dB.

The performance of the cryostat exceeds the requirements for operation (see Table 1), reaching a base temperature of $265\,\hbox {mK}$ on the $^3\hbox {He}$ cold head for over 32 h. A full cooldown from room temperature to base temperature takes about 14 h. In the next section, we will discuss the filter design and geometrical design in detail.

## Detailed System Design

### Spectral Filtering

The spectral window accessible for measurement in the cryostat is in the first order a low pass with cutoff at $f_c \approx 950$ GHz in order to include the atmospheric windows accessible from high altitude sites such as the Atacama desert [9]. The filter stack in this cryostat consists of four types of filters. A strong focus in the design is to avoid unnecessary filter heating, which has been shown to be a major issue for large filters [10]. The rejection better than $10^{6}$ at high frequencies is necessary to fully suppress the contribution of this regime’s blackbody spectrum to the total thermal radiation compared to the power in the band of interest.

The first filter mounted in the cryostat is a thermal shader (QMC Instruments Ltd.) mounted on 300 K vacuum can, but which is in radiative balance. It is transparent in-band and reflects power in the near-infrared.

It is transparent in-band and reflects power in the near-infrared. The first filter mounted in the cryostat is a thermal shader (QMC Instruments Ltd.) mounted on 300 K vacuum can but which is in radiative balance. It is transparent in-band and reflects power in the near-infrared.

**Table 1:** Operating temperatures *T* and cooling power $P_\mathrm{cool}$ for all stages of the cryostat in addition to the calculated radiative and conductive thermal loads as well as the measured total thermal load from the optical access

Stage	*T* (K)	$P_\mathrm{cool}$	$P_\mathrm{load}$ radiative	$P_\mathrm{load}$ conductive	$P_\mathrm{load}$ measured
50 K (PT)	39.5	$31.5\,\hbox {W} \,@\, 45\,\hbox {K}$	$17\,\hbox {W}$	–	–
3 K (PT)	2.85	$0.9\,\hbox {W}\, @ \,4.2\,\hbox {K}$	$217\, \upmu \hbox {W}$	–	–
1 K ($^4\hbox {He}$)	0.81	$250\, \upmu \hbox {W}\, @ \,0.85\,\hbox {K}$	$9.3\, \upmu \hbox {W}$	$0.6\, \upmu \hbox {W}$	$< 50\, \upmu \hbox {W}$
Sub-K ($^3\hbox {He}$)	0.265	$1\, \mu \hbox {W}\, @ \, 240\,\hbox {mK}$	$4.4\, \upmu \hbox {W}$	$16\,\hbox {nW}$	$6.0\, \upmu \hbox {W}$

The conductive load is dominated by wiring as the high vacuum in the cryostat ($P \approx 10^{-7}\ldots 10^{-10}\,\hbox {mbar}$) suppresses gas conduction. Pulse tube stages are denoted with ‘PT’, while $^4\hbox {He}$ and $^3\hbox {He}$ are the sorption coolers buffer and cold head respectively

**Fig. 2:**
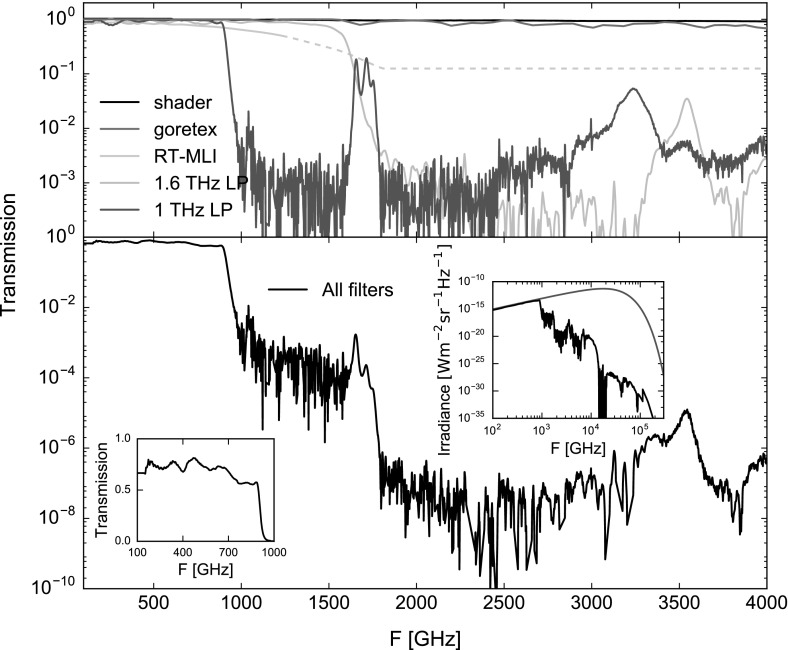
Transmission curves of the individual filters are shown in the top. At high frequencies, marked by the dotted line, the transmission curve shown for RT-MLI needs be treated as absorbing material in radiative balance. The bottom graph shows the total coherent transmission of the filter stack, which provides rejection better than $10^{6}$ at frequencies above 2 THz. Two insets show the transmission in the accessible spectral window on a linear scale (left) and the reduction of the spectral flux due to the filter stack for a $300\,\hbox {K}$ blackbody (right), where the reduction at high frequencies is dominated by the goretex sheets (Color figure online)

The second filter is the RT-MLI (radio-transparent multi-layer insulation) [8] located at the outward facing side of the 50 K window. RT-MLI is a stack of $N=7$ thin layers of styrofoam, which is transparent up to 1 THz and behaves as a diffusive absorber at higher frequencies. The individual layers of styrofoam have a uniform temperature profile and are in radiative balance, leading to thermal radiation *q* through the stack that is inversely proportional to the number of layers *N* plus one:

1 $$\begin{aligned} q \propto \frac{1}{N+1} \end{aligned}$$

Importantly, the RT-MLI reduces the broad-band infrared loading without relying on conductive cooling of the filters. For our design (see Fig. 2), the radiative balance of the RT-MLI stack is approximated by setting the minimum transmission at high frequencies, where the scattering nature of styrofoam dominates, to 1/8 as predicted by Eq. (1).

The third type of filters consists of a 1-mm- and a 2.8-mm-thin goretex sheets which are absorbtive at infrared and particularly optical frequencies. They further complement the RT-MLI by blocking optical and reducing infrared radiation from the laboratory environment.

The final type of filters is commercial low-pass metal mesh filters [10] with sharp cutoff frequencies given by the design of the metal mesh. For this cryostat, a $1.6\,\hbox {THz}$ low-pass filter is located on the 50 K stage and a $1\,\hbox {THz}$ low-pass filter is located on the 3 K stage.

The 1 K stage of the system is aperture in which we can place band-pass filters. They reduce the load on the cold stage significantly and are intended for specific detector measurements. They are not needed for cryostat operation and not further considered here.

In the implemented design, we use the thermal shader, RT-MLI and goretex to reduce the thermal loading on the lowest temperature stages. This reduces possible filter heating on the metal mesh filters at 50 K and 3 K. Additionally, the small diameter of the metal mesh filters allows some conductive cooling via the mounting structure, further reducing the problem [10]. The stepped shape of the 50 K window is a direct result of this consideration, reducing the diameter $d_{50k} = 72\,\hbox {mm}$ of the goretex and low-pass filter by a factor of 0.6 compared to the RT-MLI located on the same stage.

### Geometric Throughput

The far-infrared access to the cold stage of the system is designed by minimizing the loading of the cold stage and 1 K stage while maintaining a large opening angle. Importantly, we found during testing that the $^4\hbox {He}$ stage of our sorption cooler is limiting the hold time. Hence, our goal is to minimize the loading power on both stages of the sorption cooler. A python program was developed to quickly simulate the thermal power on all stages for various geometries using a first-order approximation of the geometric throughput integral, which underestimates the true value of the integral.

**Fig. 3:**
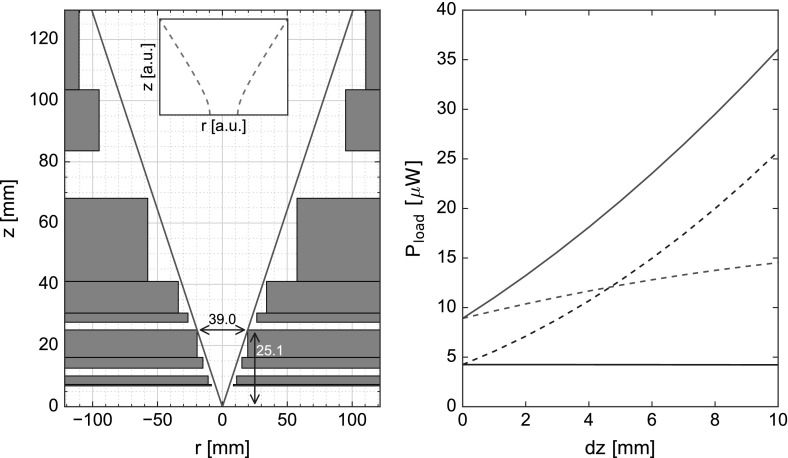
On the left, the geometry of the final cryostat design is shown as it is implemented in the geometric throughput simulation. The (0,0) position corresponds to the antenna on the cold stage, and the shields (1 K, 3 K, 50 K and vacuum can) are shown in gray. The maximum opening angle is shown in red, and the dimensions for the limiting aperture at the 3 K stage are shown in blue. The inset qualitatively shows the shape of a Gaussian beam. The graph on the right shows two cases for the thermal load on the two stages of the sorption cooler ($^4\hbox {He}$ in red and $^3\hbox {He}$ in blue) as a function of distance *dz* ($dz = 0\,\hbox {mm}$ indicating the actual position in the cryostat) while keeping a constant opening angle. The dashed lines show the increase in power if only the detector assembly is moved inwards, and solid lines show the case if both the 1 K stage and detector assembly are moved inward while maintaining equidistance with each other (Color figure online)

For the simulations, the vacuum window is assumed as a perfect blackbody of $T = 300\,\hbox {K}$, while all radiation shields are perfect absorbers and the filters only transmissive using the transmission curves as shown in Fig. 2. The effects from filter heating on the total thermal load of the sorption cooler stages are assumed to be negligible due to the design of the filter stack. Additionally, reflections are neglected, as all surfaces around the windows are coated with IR absorber, consisting of black Stycast 2850FT with $1\,\hbox {mm}$ SiC grains [11].

We design the apertures in the vacuum shield, 50 K shield and 3 K shield to accommodate the previously described filter stack and allow for the vertical assembly of the cryostat. The limiting aperture determining the opening angle is located on the outer edge of the 3 K stage, which exploits the large cooling power of the pulse tube (see Table 1). We position the detector assembly and 1 K stage as close as possible to the 3 K stage, as this results in the lowest possible loading on these two stages (see Fig. 3). This also leads to small aperture diameters at the higher temperature stages (for a fixed opening angle), which enables the use of smaller metal mesh filters, reducing the effects of filter heating. Additionally, it limits the size of the required vacuum window. The windows are designed in step shapes with a large outer aperture and a small inner aperture (see Fig. 3) to reduce loading on the colder stages while avoiding grazing incident radiation, as SiC in Stycast has a strongly reduced absorption efficiency at high incident angles [11].

To determine thermal loading of the cold stage from the measured temperature, a load calibration $P(T_\mathrm{stage})$ was created using a resistive heater mounted on the stage under dark conditions. Temperature measurements were then taken with an absorber mounted at the detector position with open windows. Simulation results are in good agreement with the hereby obtained measurements, predicting a thermal load on the cold stage of $P_{l,r,\mathrm{calc}} = 4.4\, \upmu \hbox {W}$ compared to the measured $P_{l,\mathrm{meas}} = 6.0\, \upmu \hbox {W}$.

## Conclusion

We have designed a cryostat for beam pattern measurements in the sub-mm at large angles up to $\theta = 37.8^\circ$. A first-order thermal radiation model was developed and used to design the apertures and their positions on the cryostat thermal stages. The model predicts a load on the cold stage of $4.4\, \upmu \hbox {W}$, quite close to the measured value of $6\, \upmu \hbox {W}$ and well within the expected accuracy of the model. A filter stack combining various filter types is designed to improve rejection of out-of-band radiation and reduce filter heating. Operation of the cryostat shows excellent performance, reaching a base temperature of $265\,\hbox {mK}$ in 14 h and hold time longer than 32 h.
